# Risk‐Benefit Considerations in Deep Brain Stimulation Discontinuation for Late‐Stage Parkinson's Disease

**DOI:** 10.1002/mdc3.70558

**Published:** 2026-02-13

**Authors:** Pietro Antenucci, Andrea Gozzi, Fabiana Colucci, Federica Pes, Jay Guido Capone, Alba Scerrati, Michele Alessandro Cavallo, Maura Pugliatti, Daniela Gragnaniello, Mariachiara Sensi

**Affiliations:** ^1^ Unit of Clinical Neurology, Interdistrict Department of Neuroscience and Rehabilitation University of Ferrara Ferrara Italy; ^2^ Unit of Neurology, Interdistrict Department of Neuroscience Sant'Anna University‐Hospital Ferrara Italy; ^3^ Parkinson and Movement Disorders Unit, Department of Clinical Neurosciences, Fondazione IRCCS Istituto Neurologico Carlo Besta Milan Italy; ^4^ Neurophysiology Unit, Interdistrict Department of Neuroscience Sant'Anna University‐Hospital Ferrara Italy; ^5^ Unit of Neurosurgery, Arcispedale Sant'Anna Hospital Ferrara Italy; ^6^ Department of Translational Medicine University of Ferrara Ferrara Italy

**Keywords:** decision‐making, deep brain stimulation, late stage, Parkinson's disease, risk–benefit assessment

## Abstract

**Background:**

Management of deep brain stimulation (DBS) in late‐stage Parkinson's disease (LSPD) remains challenging, particularly when deciding whether to continue or discontinue stimulation, and evidence on risk–benefit considerations is limited.

**Objectives:**

To identify key factors to improve decision‐making in DBS management for LSPD patients.

**Methods:**

We retrospectively analyzed demographic, clinical and stimulation parameters in LSPD patients (Hoehn and Yahr ≥4; Schwab and England ≤50) who either maintained best medical therapy (BMT) or required unscheduled device‐aided therapy (DAT) implantation up to 1 year after DBS discontinuation.

**Results:**

From 2005 to 2022, 94 patients with bilateral subthalamic nucleus DBS were reviewed and among the 31 patients who have transitioned to LSPD, 15 patients remained on BMT, while 10 required rescue DAT (6 unscheduled implantable pulse generator replacements and 4 Levodopa‐Carbidopa Intestinal Gel) within 3 months after discontinuation. Significant differences were observed in years of DBS (12.4 vs. 8.5), modified Falls Efficacy Scale (12.5 vs. 21.2), and months since the last parameter adjustment (30.3 vs. 23.2), with a trend toward less ΔMDS‐UPDRS III worsening after stimulation was switched off (7.6 vs. 10.9). Longer DBS duration was inversely associated with rescue DAT (OR 0.529; 95% CI, 0.284–0.986), with a cutoff of 10.5 years.

**Conclusion:**

In selected LSPD patients, a transition from DBS to BMT alone can be attempted with long‐term stability, whereas in others a more conservative approach is advisable, and stimulation should be continued. Clinical, therapeutic, and care‐related factors should guide decisions when discontinuation is being considered.

Parkinson's disease (PD) is a progressive neurodegenerative disorder in which motor complications—motor fluctuations, and dyskinesias—typically occurred largely due to the gradual narrowing of levodopa's therapeutic window.^1^ Patients experience discomfort and therapeutic strategies are needed to improve complications and subsequent quality of life. When optimization of oral therapy is insufficient to achieve satisfactory symptom control, treatment options that may be considered include device‐aided therapies (DAT), such as deep brain stimulation (DBS) or infusion therapies.^2^

Among DAT options, subthalamic nucleus DBS (STN‐DBS) is well established as an effective intervention^3^, ^4^—particularly in patients whose symptoms remain levodopa‐responsive^5^ and its indications include improvement of motor fluctuations in moderate‐advanced stage of the disease with more recent indication in earlier phases in selected patients with initial, milder motor fluctuations.^2^ However, as the disease progresses, the response to levodopa diminishes and axial and non‐motor symptoms—such as cognitive decline, hallucinations, autonomic dysfunction, speech and swallowing difficulties, and postural instability—become predominant.^6^ These features mark the transition to *late‐stage PD* (LSPD), a phase in which disability for both patients and caregivers is often driven more by these refractory symptoms than by motor complications alone.^7^

Currently, no consensus exists on the optimal management of STN‐DBS in patients who have transitioned to LSPD. Evidence indicates that STN‐DBS may confer sustained benefits on levodopa‐responsive motor symptoms (tremor, bradykinesia, rigidity),^8^, ^9^, ^10^ while worsening certain motor and non‐motor symptoms (axial symptoms, freezing of gait (FOG), orthostatic hypotension) as late‐stage features progress.^3^, ^7^, ^8^, ^11^ For this reason, a stimulation challenge test and regular follow‐up to assess motor function and quality of life^10^ have been proposed as the most effective strategies when considering DBS discontinuation in LSPD. In practice, this approach is limited by patient complexity, care burden,^12^ and possible persistence of DBS effects in poor responders, sometimes requiring reactivation.^13^, ^14^, ^15^ Furthermore, in patients with non‐rechargeable implantable pulse generators (nr‐IPGs), battery depletion necessitates surgical replacement approximately every 4–5 years. Therefore, discontinuation of stimulation and transition to best medical therapy (BMT), following shared decision‐making among clinicians, patients, and caregivers, is not uncommon and generally considered safe, with life‐threatening withdrawal complications reported in a small minority of cases.^16^

On these grounds, DBS management in LSPD remains debated, and optimizing withdrawal could reduce patient risks and discomfort,^16^ may provide economic benefits given DATs’ cost‐effectiveness,^17^, ^18^ and addresses ethical concerns in cognitively impaired patients, supporting informed advanced directives.^19^, ^20^ Evidence directly comparing LSPD patients who continue with BMT alone versus those requiring additional DAT after DBS discontinuation is still lacking and may provide valuable insights on the topic.

## Aim of the Study

Analysis of the clinical and therapeutic management of DBS discontinuation in a cohort of patients with LSPD, and evaluation of the factors determining either successful continuation with BMT alone or the subsequent need for additional rescue DAT.

## Methods

### Study Design

Monocentric, observational, retrospective cohort study conducted at the Movement Disorders Clinic, Neurology Department, Arcispedale Sant'Anna di Cona, Ferrara. Participants or their legal representatives provided informed consent, and the study is part of a broader protocol approved by the local ethics committee (489/2023/Oss/AOUFe). The study was conducted in accordance with the ethical standards of the 1964 Declaration of Helsinki and its later amendments.

### Recruitment and Outcome Measures

All DBS patients with LSPD followed at our center who underwent DBS device discontinuation and had at least 1 year of complete follow‐up were considered eligible and included in the study. Late‐stage disease was defined according to the literature as Hoehn and Yahr (H&Y) stage ≥4 and Schwab and England scale ≤50.^21^ Within this cohort, patients who, 1 year after discontinuation either continued successfully on BMT alone or required an additional DAT (Implantable Pulse Generator (IPG) replacement, Levodopa‐Carbidopa Intestinal Gel (LCIG), or apomorphine pump) were identified and compared.

### Data Collection

Demographics (age, sex), Clinical Features (disease duration, years of DBS, Levodopa Equivalent Daily Dose (LEDD), H&Y), Motor (ON medication/ON stimulation and ON Medication/OFF stimulation Movement Disorders Society‐Unified Parkinson's Disease Rating Scale (MDS‐UPDRS) part III), Non‐Motor Symptoms Scale (NMSS), and cognitive status (Mini‐Mental State Examination (MMSE), Charlson Comorbidity Index (CCI), modified Falls Efficacy Scale (mFES), Freezing of Gait questionnaire (FOGq)), and stimulation‐related parameters (IPG type, battery, substitutions and parameters of stimulation) were collected from discharge letters, outpatient clinical reports and healthcare reports at the evaluation preceding the discontinuation. The residual effect of stimulation on motor symptoms was assessed at the time of DBS discontinuation as the difference between the OFF‐stimulation/ON‐medication MDS‐UPDRS III and the ON‐stimulation/ON‐medication MDS‐UPDRS III scores (ΔMDS‐UPDRS III). LEDD was assessed at the time of DBS implantation (T0), at parameters stabilization after about 1 year from DBS implant (T1), at the time of suspension (T2) and up to 1 year of follow‐up after DBS discontinuation (T3). When only UPDRS was available, it was converted to MDS‐UPDRS according to the literature.^22^

### Statistical Analysis

Data are presented as measures of central tendency and dispersion (mean ± standard deviation) for continuous variables, and as absolute frequencies (counts or percentages) for categorical variables. Categorical variables were compared using the chi‐square test or Fisher's exact test, while continuous variables were compared using either the t‐test or the Mann–Whitney *U* test, depending on their distribution as assessed by the Shapiro–Wilk test. Group differences were further explored using analysis of covariance (ANCOVA). In addition, a logistic regression analysis was performed to simultaneously evaluate the influence of multiple independent variables on the dependent variable (oral therapy alone vs. additional device‐aided therapy), in order to isolate the unique contribution of each potential predictor while controlling for the others. To assess the discriminative ability of the variable of interest, a receiver operating characteristic (ROC) curve was constructed based on a univariate or multivariate logistic regression model depending on the context, and the area under the curve (AUC) was calculated. The optimal cut‐off point was determined using the Youden Index. All analyses were conducted using SPSS software (IBM, version 25), with statistical significance set at *p* < 0.05.

## Results

Between 2005 and 2022, 94 PD patients with bilateral STN‐DBS had been followed at our center. Among them, we identified 31 patients who have transitioned to LSPD. Two were lost to follow‐up and four still have the stimulation activated at the moment of the data collection, while 25 experienced DBS discontinuation and were considered eligible and included in the study. All patients had a nr‐IPG and for all the group the decision to discontinue stimulation was made concurrently with the need to replace the battery, based on the clinician's recommendation and/or at the patient's and family members' will, following an unfavorable empirically assessed risk/benefit balance considering the surgical procedure, the potential clinical benefit of continuing stimulation (including residual improvement in motor symptoms, control of fluctuations and dyskinesias, presence of DBS‐induced axial symptoms, and overall impact of cognitive and non‐motor symptoms), and the level of care requirements.

At the time of DBS discontinuation, the mean age was 73.7 ± 5.2 years, with a mean disease duration of 23.6 ± 5.5 years and a mean DBS treatment duration of 10.8 ± 3.2 years. Fifteen patients successfully completed 1 year on oral BMT alone (Group 0), while 10 patients required the initiation of a new DAT for advanced‐complicated PD phase, within 1 year after DBS discontinuation (Group 1). Among this group, six required a not scheduled IPG replacement and four LCIG after a mean of, respectively, 18.2 ± 13.1 and 35.3 ± 20.1 days from the discontinuation. The main causes leading to re‐consider DAT following DBS discontinuation were 3: the occurrence of a rapid subacute worsening of akinetic‐rigid symptoms unresponsive to increased oral levodopa (4, IPG replacement), the presence of a sustained good response to stimulation on motor symptoms despite a marked worsening of levodopa‐related cognitive and neuropsychiatric symptom (2, IPG replacement), and a significative response to dopaminergic oral therapy in the presence of predominant axial symptoms (particularly dysphagia) (4, LCIG).

Group 1 showed a higher, though non‐significant, baseline LEDD at DBS implantation (1192.9 ± 248.7 vs. 1450.0 ± 309.3 mg, *p* = 0.070). Overall LEDD reduction from T0 to T2 was comparable between groups (−416.1 ± 154.9 vs. −575.0 ± 387.5 mg, *p* = 0.177), greater in Group 0 between T0 and T1 (25.5% vs. 16.7%), whereas between T1 and T2 in Group 1 (14.4% vs. 26.9%). At T3, no significant differences were observed (803.4 ± 189.4 mg vs. 1009.0 ± 402.4 mg, *p* = 0.097).

Clinical and demographic characteristics of the two groups at the time of DBS discontinuation are reported in Table 1. Significant differences (Group 0 vs. Group 1) were present for years of DBS therapy (12.3 ± 3.1 vs. 8.6 ± 1.9, *p* = 0.003), the assessment of subjective fall risk (mFES 12.9 ± 7.7 vs. 20.6 ± 21.1, *p* = 0.045) and in the number of months since the last parameter adjustment prior to device deactivation (30.7 ± 12.3 vs. 22.5 ± 5.8, *p* = 0.035). A differential trend was instead observed for ΔMDS‐UPDRS III at the consultation prior to DBS discontinuation (7.6 ± 4.0 vs. 10.9 ± 4.5, *p* = 0.065).

**TABLE 1:** Characteristics of patients who discontinued DBS treatment

	Patients with oral medication therapy after 1 year (N = 15, Group 0)	Patients with advanced device‐aided therapy after 1 year (N = 10, Group 1)	*p*‐value
Age (years)	73.7 ± 6.0	73.8 ± 6.1	0.935
Sex (M/F)	5/10	6/4	0.188
Duration of disease (years)	23.9 ± 5.2	23.1 ± 6.0	0.739
Years of DBS (years)	12.3 ± 3.1	8.6 ± 1.9	0.003^a^
IPG Type (implant)			0.607
Medtronic‐Kinetra	6	7	
Medtronic‐Activa PC	6	2	
St. Jude Medical‐Libra	2	1	
Abbott‐Libra	1	0	
Substitution (n)	2.0 ± 0.7	1.3 ± 0.5	0.119
MMSE	16.1 ± 3.7	14.4 ± 3.4	0.238
ON/ON MDS‐UPDRS III	44.8 ± 8.4	49.6 ± 10.2	0.210
LEDD (mg)	758.3 ± 197.2	895.0 ± 276.3	0.162
H&Y	4.3 ± 0.5	4.3 ± 0.5	0.863
S&E scale	28.0 ± 16.6	28.0 ± 9.2	1.000
NMSS	114.5 ± 40.3	121.1 ± 42.0	0.695
CCI	4.3 ± 1.4	3.8 ± 1.0	0.374
mFES	12.9 ± 7.7	20.6 ± 21.1	0.045^a^
FOGq	12.2 ± 4.4	9.3 ± 3.1	0.144
Stimulation parameters			
Amplitude (mA)	4.0 ± 0.8	3.5 ± 0.6	0.079
Width (μsec)	64.8 ± 9.1	62.2 ± 4.2	0.144
Frequency (Hz)	106.8 ± 26.4	125.0 ± 15.8	0.765
Last time parameters adjustment (months)	30.7 ± 12.3	22.5 ± 5.8	0.035^a^
Δ MDS‐UPDRS III	7.6 ± 4.0	10.9 ± 4.5	0.065^b^

*Note*: Data are presented as mean ± standard deviation and numbers.

Abbreviations: AR, Akinetic‐Rigid; CCI, Charlson comorbidity index; DBS, deep brain stimulation; F, female; FOGq, freezing of gait questionnaire; H&Y, Hoehn and Yahr Scale; Hz, Hertz; IPG, implantable pulse generator; LEDD, levodopa equivalent daily dose; M, male; mA, milliAmpere; mFES = modified falls efficacy scale; mg, milligrams; MMSE, mini‐mental state examination; N, number; NMSS, Non‐Motor Severity Scale; ON/ON MDS‐UPDRS III, ON stimulation/ON medication movement disorder society‐Unified Parkinson's disease rating scale III; S&E scale, Schwab and England scale; TD, Tremor dominant; μsec, microseconds; ΔMDS‐UPDRS III, OFF Stimulation/ON Medication MDS‐UPDRS III‐ON Stimulation/ON Medication MDS‐UPDRS III.

^a^ Statistically significant.

^b^ Statistical trend.

After adjusting for age, disease duration, motor severity (ON/ON MDS‐UPDRS III), and LEDD, significant differences persisted for years of DBS therapy (12.4 vs. 8.4, *p* = 0.003), mFES (12.3 vs. 21.5, *p* = 0.029), and months since last parameter adjustment (30.7 vs. 22.5, *p* = 0.026), with age and disease duration significantly contributing to the latter (*F* = 4.531, *p* = 0.047, *F* = 13.441, *p* = 0.002). A trend toward divergence was also noted for ΔMDS‐UPDRS III, although it did not reach statistical significance (7.4 vs. 11.2, *p* = 0.056). ANCOVA results are shown in Table 2.

**TABLE 2:** Expected means, confidence intervals, and *p*‐values adjusted of the characteristics of the two groups, controlling for covariates including age, disease duration, motor symptom severity and Levodopa equivalent daily dose (LEDD) at the time of DBS withdrawal (ANCOVA).

	Patients with oral medication therapy after 1 year (N = 15, Group 0)	Patients with advanced device‐aided therapy after 1 year (N = 10, Group 1)	*p*‐value
Years of DBS (y)	12.4 (10.9–14.0)	8.4 (6.5–10.3)	0.003^a^
	Age (y)	1.839	0.191
	Duration of disease (y)	0.881	0.360
	ON/ON MDS‐UPDRS III	0.745	0.399
	LEDD (mg)	0.087	0.771
mFES	12.3 (7.4–17.2)	21.5 (15.3–27.6)	0.029^a^
	Age (y)	2.218	0.153
	Duration of disease (y)	0.198	0.662
	ON/ON MDS‐UPDRS III	0.998	0.330
	LEDD (mg)	0.197	0.662
Last time parameters adjustment (months)	30.7 (26.4–35.0)	22.5 (17.0–27.9)	0.026^a^
	Age (y)	4.531	0.047^a^
	Duration of disease (y)	13.441	0.002^a^
	ON/ON MDS‐UPDRS III	0.284	0.600
	LEDD (mg)	1.655	0.214
ΔMDS‐UPDRS III	7.4 (5.1–9.8)	11.2 (8.3–14.0)	0.056^b^
	Age (y)	0.097	0.758
	Duration of disease (y)	3.212	0.089
	ON/ON MDS‐UPDRS III	0.259	0.616
	LEDD (mg)	0.804	0.381

*Note*: The effect of the covariates is quantified using the F statistic from the ANCOVA model.

Abbreviations: DBS, deep brain stimulation; mFES, modified Falls Efficacy Scale; N, number; ON/ON MDS‐UPDRS III, ON stimulation/ON medication movement disorder society‐Unified Parkinson's disease rating scale III; y, years; ΔMDS‐UPDRS III, OFF stimulation/ON medication MDS‐UPDRS III‐ON stimulation/ON medication MDS‐UPDRS III.

^a^ Statistically significant.

^b^ Statistical trend.

In the logistic regression model, only years of DBS therapy were significantly associated with the outcome (Assignment to Group 1—need for an additional DAT after DBS discontinuation, *B* = −0.636, *p* = 0.045), with an odds ratio (OR) of 0.529 (95% CI: 0.284–0.986), indicating a protective effect as every additional year of DBS reduces the odds of the outcome by approximately 47%. mFES scores (*p* = 0.413), months since the last parameter adjustment (*p* = 0.410), and ΔMDS‐UPDRS III (*p* = 0.082) were not significantly associated with the outcome, although the latter showed a trend toward significance (OR = 1.321, 95% CI: 0.965–1.808). Logistic regression model is displayed in Table 3.

**TABLE 3:** Results of the logistic regression analysis predicting the probability of being in Group 1, ie, requiring additional advanced aided therapy after DBS discontinuation.

Predictor	*β* (beta)	OR	95% CI	*p*‐value
Years of DBS (y)	−0.636	0.53	0.28–0.98	0.045*
mFES	0.097	1.10	0.87–1.39	0.413
Last time parameters adjustment (months)	−0.075	0.93	0.78–1.11	0.410
ΔMDS‐UPDRS III	0.278	1.32	0.97–1.81	0.082

*Note*: The table reports regression coefficients (β), odds ratios (OR) with 95% confidence intervals (CI), and *p*‐values for each predictor in the model. Significant predictors are indicated (*p < 0.05).

Abbreviations: CI, confidence interval; DBS, deep brain stimulation; mFES, modified Falls Efficacy Scale; OR, odds ratio; y, years; ΔMDS‐UPDRS III, OFF stimulation/ON medication MDS‐UPDRS III‐ON stimulation/ON medication MDS‐UPDRS III.

**FIG. 1:**
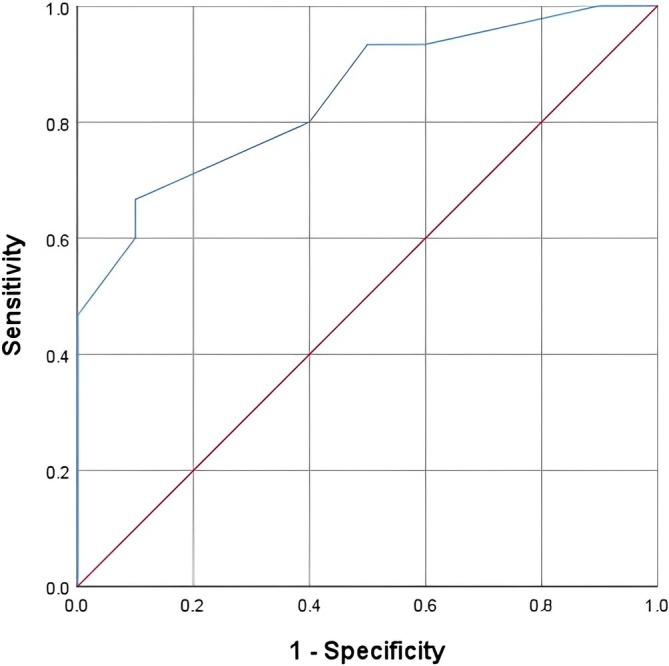
Receiver operating characteristic (ROC) curve for the univariate predictor “years of DBS treatment” in relation to belonging to Group 0 (continuation with oral therapy alone following DBS discontinuation). The model demonstrated good discriminatory capacity with an area under the curve (AUC) of 0.843 (95% CI: 0.692–0.995). The optimal cutoff point determined by the Youden index was 10.5 years of DBS treatment, corresponding to an index value of 0.567. At this threshold, the sensitivity and specificity were 66.7% and 90.0%, respectively.

A univariate ROC curve was built applying years of DBS therapy to predict the risk of requiring advanced treatment following DBS deactivation. The outcome was coded as 0 for no need of advanced therapy, indicating an inverse relationship whereby longer DBS duration corresponds to a reduced likelihood of needing further intervention. The model demonstrated good discriminatory power, with an area under the curve (AUC) of 0.843. The optimal cut‐off identified by the Youden index was 10.5 years of DBS, yielding an index value of 0.567, sensitivity and specificity were 66.7% and 90.0% at this threshold. These findings underscore the protective role of extended DBS duration in reducing the risk of subsequent advanced treatment after device deactivation. ROC curve is presented in Figure 1.

## Discussion

“Should we consider deep brain stimulation discontinuation in late‐stage Parkinson's disease?” is an extremely timely question, highly relevant for clinicians managing advanced therapies for PD.^10^ A good response to DBS is still observed in approximately 80% of LSPD patients, while about 20% showed only limited benefit (24% in our cohort applying the same proposed criteria (ie, improvement in MDS‐UPDRS Part III < 10%)), raising the question of whether discontinuation of stimulation should be considered in this subgroup.^10^ So, an equally critical question that needs to be addressed is: “If so, when and in which patients?”.

Consistent with previous reports,^10^, ^16^ our results show that a milder stimulation efficacy and a longer period of stimulation stability before discontinuation, could predict effective maintenance on BMT alone in LSPD after DBS withdrawal.

It could be hypothesized that, in this small group of patients in whom DBS‐resistant non‐motor symptoms prevail, disability related to motor complications tends to be less significant and less debilitating.^23^ Moreover, in some cases in which chronic stimulation itself has caused adverse effects (such as axial symptoms or dysarthria), DBS loses its primary indication, and its discontinuation does not result in a significant rebound of the residual component of motor fluctuations, which can be effectively controlled by medical therapy alone.

Unlike our findings, previous studies have linked a long history of DBS treatment to withdrawal syndrome requiring reimplantation.^16^ However, the aforementioned mainly compared patients with ~10 years of DBS to those with ~3 years, in whom discontinuation appeared safer.

Taken together, these data suggest that PD patients’ response to DBS may progress through at least three phases: an initial phase (~3–5 years) with clear benefit and safe discontinuation; a middle phase (5–10 years) where plastic changes and reduced dopamine sensitivity make discontinuation potentially risky; and a late phase (>10 years), as probably in our PD population, dominated by non‐motor symptoms not responsive to dopaminergic therapy, with disease progression outweighing residual DBS effects.^16^, ^24^, ^25^ However, our and previous data are insufficient to consider DBS duration as a decisive variable per se in the decision‐making algorithm. The marked heterogeneity of PD further complicates long‐term outcome prediction and may account for the variability reported in other studies.

The greatest increase in LEDD after DBS discontinuation was observed in the group initiating DAT (12.7% vs. 6.0%). As expected^26^ within Group 1, the largest LEDD increase occurred in the LCIG subgroup (+38.1%), whereas the subgroup undergoing nr‐IPG reimplantation showed a relative, non‐significant LEDD reduction over the same period (−5.6%).

Notably, the need for rescue therapy after DBS discontinuation occurred within 3 months for all patients, earlier for IPG replacements (18.5 days) and later for LCIG (35.3 days). This suggests that patients able to switch to BMT in the short‐ to mid‐term are likely to maintain this outcome over long‐term follow‐up, whereas those requiring DAT after discontinuation present distinct complication profiles with heterogeneous timing, particularly early onset of motor symptom worsening responsive to stimulation and levodopa‐related adverse effects.

Considering the impact of DBS on axial symptoms, our results suggest that assessing fall‐related burden via the mFES can guide DBS withdrawal. Patients with higher burden may fare better with BMT alone, while those with preserved postural stability may benefit more from DAT, even in late‐stage disease. However, this interpretation is limited by the subjective nature of the mFES and the heterogeneous etiopathogenesis of falls in LSPD,^27^ as different mechanisms may influence adaptation to device discontinuation. Supporting this, increased falls may relate to the presence of FOG, potentially influenced by STN‐DBS stimulation, whereas BMT could provide improvement. Although no significant differences in FOGq were observed in our cohort, this aspect warrants longitudinal investigation.

This study has several limitations, including its retrospective, single‐center design, which limits the sample size and the reliance on scales that assess functional impact subjectively, without standardized measures. Comprehensive cognitive assessments at the time of DBS discontinuation, as well as evaluations of quality of life and caregiver burden, were not available. Accordingly, these findings should be interpreted with caution. A multidimensional approach remains essential when considering DBS discontinuation, taking into account the risk–cost–benefit profile, patient preferences, and caregiver need. Future studies should include comprehensive clinical assessments of post‐implantation responses to stimulation and, ideally, larger patient cohorts to reduce the risk of overfitting and more reliably identify potential predictors of outcomes in LSPD, thereby confirming the preliminary findings reported here.

In conclusion, managing DBS in LSPD is challenging particularly when considering whether to continue stimulation. While efficacy of DBS on residual motor fluctuation is likely a key factor in the clinician's risk–benefit evaluation, other aspects, such as progressive prevalence of non‐motor symptoms not responsive to DBS such as cognitive deterioration, axial symptoms and falls, should also be considered and, as in our experience the long DBS duration should not be considered invariably associated with withdrawal syndrome and the option of DBS cessation could be considered in these patients.

A multidimensional approach involving both patients and caregivers is likely essential, though further studies are needed.

## Author Roles

(1) Research project: A. Conception, B. Organization, C. Execution; (2) Statistical Analysis: A. Design, B. Execution, C. Review and Critique; (3) Manuscript: A. Writing of the first draft, B. Review and Critique.

P.A.: 1A, 1B, 1C, 2A, 2B, 3A.

A.G.: 1C, 2B.

F.C.: 1A, 2C.

F.P.: 1B, 1C.

J.G.C.: 3B.

A.S.: 1A, 3B.

M.A.C.: 3B.

M.P.: 2C, 3B.

D.G.: 3B.

M.S.: 1A, 3B.

## Disclosures

**Ethical Compliance Statement:** We confirm that we have read the Journa’'s position on issues involved in ethical publication and affirm that this work is consistent with those guidelines. Participants or their legal representatives provided informed consent, and the study is part of a broader protocol approved by the local ethics committee “Comitato Etico Area Vasta Emilia Centro CE‐AVEC sez. Ferrara, ref. number 489/2023/Oss/AOUFe.”

**Funding Sources and Conflicts of Interest:** PA, AS, and MS conceived the study and drafted the initial manuscript. PA, AG, and FP collected clinical data and performed the statistical analysis. FC and MP supervised data collection and provided methodological support. MS, DG, AS, JGC and MAC contributed to critical revision of the manuscript and interpretation of the results. All authors have read and approved the final version of the manuscript and take responsibility for all aspects of the work.

**Financial Disclosures for the Previous 12 Months:** The authors declare that there are no additional disclosures to report.

## Financial Disclosures and Conflicts of Interest

Author disclosures are available in the Supporting Information.

## Supporting information


**Data S1** Coi_disclosure.

## Data Availability

The data that support the findings of this study are available from the corresponding author upon reasonable request.
